# A Case of Isolated Oral Secondary Syphilis

**DOI:** 10.31662/jmaj.2021-0180

**Published:** 2021-12-08

**Authors:** Kiyozumi Suzuki, Akihiro Kanzawa, Hiromasa Otsuka, Yuji Hirai

**Affiliations:** 1 Department of Emergency Room and General Medicine, Ageo Central General Hospital, Ageo, Japan; 2 Department of Infectious Disease, Tokyo Medical University Hachioji Medical Center, Tokyo, Japan

**Keywords:** secondary syphilis, *Treponema pallidum*, aphthous ulcers, isolated oral lesions

A 40-year-old man presented to our hospital with a 7-week history of oral lesions with mild pain. He was initially diagnosed with aphthous ulcers by a dentist. Skin and genital lesions were absent, but he disclosed having unprotected intercourse with a female sex worker 5 months before presentation.

Physical examination revealed irregular, whitish ulcerations on the lower lip and tongue (Figure 1 and 2). Rapid plasma reagin [titer: 1:32] and *Treponema pallidum* hemagglutination were positive, and the HIV test result was negative. Based on a reasonable incubation period, clinical oral findings, and serological tests, a diagnosis of oral secondary syphilis was made. The oral lesions resolved within 1 week of treatment with amoxicillin.

**Figure 1.**
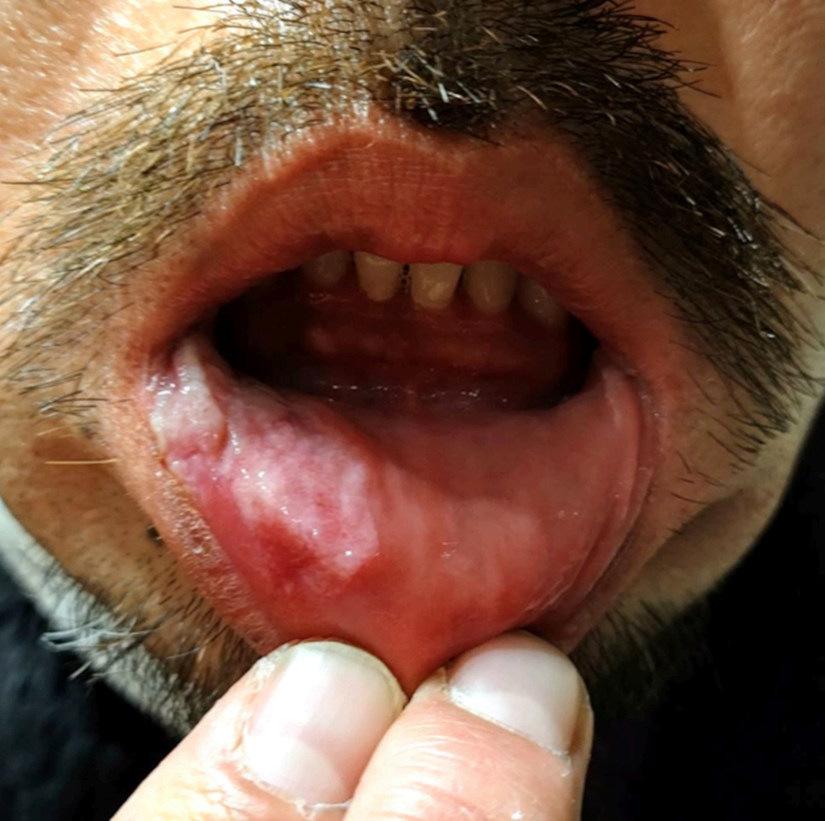
An irregular, whitish, coalescing ulcer surrounded by an erythematous area on the lower labial mucosa.

**Figure 2.**
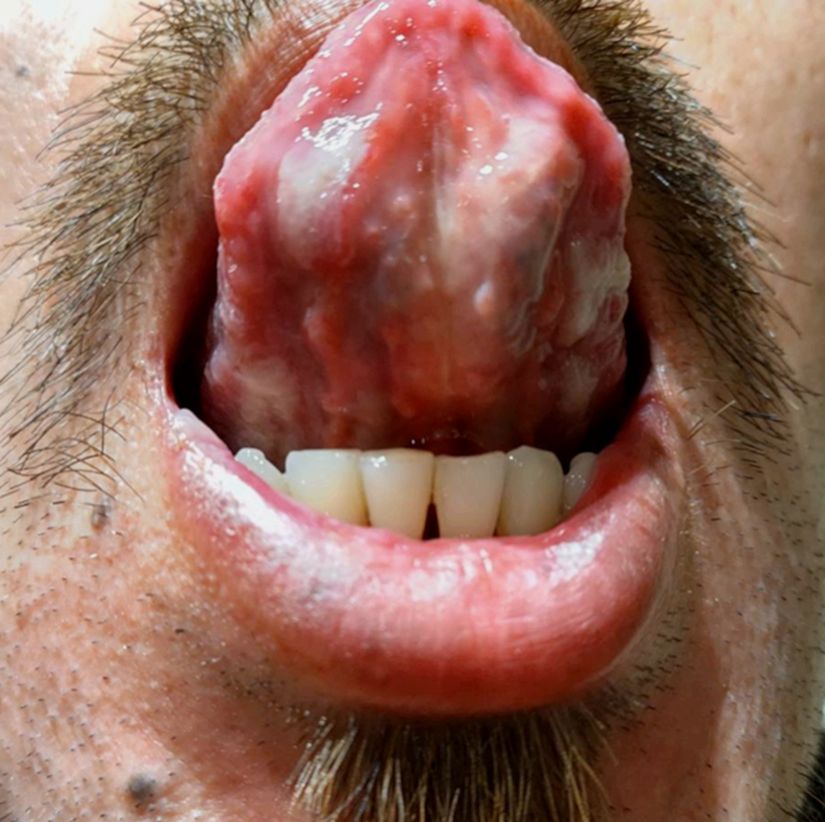
Multiple irregular whitish ulcers on the ventral surface of the tongue.

Oral lesions of primary syphilis (so called oral chancres) are typically solitary, painless, indurated ulcers that heal spontaneously within 4-5 weeks ^(1)^. In contrast, oral lesions of secondary syphilis are typically painful, multiple, and generally accompanied by cutaneous eruption ^(1)^. Isolated oral lesions have been reported to occur in only 7% of patients with secondary syphilis ^(2)^, but the condition is potentially underestimated or misdiagnosed ^(3)^. Healthcare professionals should consider oral syphilis, including the secondary stage, in the differential diagnosis of isolated oral lesions. A key to early diagnosis and treatment is confirmation of sexual history and prompt syphilis serological testing.

## Article Information

### Conflicts of Interest

None

### Author Contributions

Kiyozumi Suzuki: Writing-Original draft, Methodology

Akihiro Kanzawa: Methodology, Writing-review & editing

Hiromasa Otsuka: Methodology, Writing-review & editing

Yuji Hirai: Methodology, Writing-review & editing

All authors critically reviewed the manuscript.

### Approval by Institutional Review Board (IRB)

In this study, IRB approval was not required.

### Informed Consent

Consent was obtained from the patient for the use of images for publication.
